# Depression and the risk of coronary heart disease: a meta-analysis of prospective cohort studies

**DOI:** 10.1186/s12888-014-0371-z

**Published:** 2014-12-24

**Authors:** Yong Gan, Yanhong Gong, Xinyue Tong, Huilian Sun, Yingjie Cong, Xiaoxin Dong, Yunxia Wang, Xing Xu, Xiaoxu Yin, Jian Deng, Liqing Li, Shiyi Cao, Zuxun Lu

**Affiliations:** Department of Social Medicine and Health Management, School of Public Health, Tongji Medical College, Huazhong University of Science and Technology, No. 13 Hangkong Road, Wuhan, Hubie 430030 China; Department of Tuberculosis Control, Bao’an Chronic Disease Prevent and Cure Hospital, Shenzhen, Guangdong China; Division of Nephrology, Nanfang Hospital, Southern Medical University, Guangzhou, Guangdong China; Department of Management, School of Economics and Management, Jiangxi science and technology normal university, Nanchang, Jiangxi China

**Keywords:** Depression, Coronary heart disease, Myocardial infarction, Meta-analysis

## Abstract

**Background:**

Several systematic reviews and meta-analyses demonstrated the association between depression and the risk of coronary heart disease (CHD), but the previous reviews had some limitations. Moreover, a number of additional studies have been published since the publication of these reviews. We conducted an updated meta-analysis of prospective studies to assess the association between depression and the risk of CHD.

**Methods:**

Relevant prospective studies investigating the association between depression and CHD were retrieved from the PubMed, Embase, Web of Science search (up to April 2014) and from reviewing reference lists of obtained articles. Either a random-effects model or fixed-effects model was used to compute the pooled risk estimates when appropriate.

**Results:**

Thirty prospective cohort studies with 40 independent reports met the inclusion criteria. These groups included 893,850 participants (59,062 CHD cases) during a follow-up duration ranging from 2 to 37 years. The pooled relative risks (RRs) were 1.30 (95% CI, 1.22-1.40) for CHD and 1.30 (95% CI, 1.18-1.44) for myocardial infarction (MI). In the subgroup analysis by follow-up duration, the RR of CHD was 1.36 (95% CI, 1.24-1.49) for less than 15 years follow-up, and 1.09 (95% CI, 0.96-1.23) for equal to or more than 15 years follow-up. Potential publication bias may exist, but correction for this bias using trim-and-fill method did not alter the combined risk estimate substantially.

**Conclusions:**

The results of our meta-analysis suggest that depression is independently associated with a significantly increased risk of CHD and MI, which may have implications for CHD etiological research and psychological medicine.

**Electronic supplementary material:**

The online version of this article (doi:10.1186/s12888-014-0371-z) contains supplementary material, which is available to authorized users.

## Background

Cardiovascular disease (CVD) is now a public health crisis in both developed and developing countries. It is the leading cause of mortality and extorts heavy social and economic costs globally [1,2]. Although regular physical activity and maintenance of a healthy diet (and health weight) are probably the most crucial ways to prevent the disease [3-5], mental health status may provide an additional preventive strategy against CHD risk.

Depression is a common mental disorder, which usually causes severe disability and imposes a huge burden of disease on individuals, families and societies [6]. It is estimated that 5.8% of men and 9.5% of women will experience a depressive episode in any given year [6]. Depression is highly prevalent in the world. The average lifetime prevalence of depression has been estimated at 14.6% in high-income countries, 11.1% in low-to middle-income countries [7]. According to the estimate by World Health Organization (WHO), depression will become the second leading cause of disability-adjusted life years lost by the year 2020 [8]. During the past decades, a large body of epidemiological studies suggested that depression was associated with an increased risk of chronic diseases [9], including CHD.

Previous reviews [10-14] showed that depression has been associated with CHD, but they had some limitations. In 1987, the first meta-analysis [10] showed that depression was an risk factor for CHD, but most of the studies included in that review were cross-sectional in design [11], which maybe have more confounding bias. As we know, the prospective cohort study owned the strongest evidence in observational studies. Prospective data to exclude some possible sources of bias that may exist in retrospective data could do good to come to more definitive conclusions. Rugulies [11] showed that depression was a predictor for coronary heart disease based on cohort studies, while the review did not fully investigate other subgroups except for the type of depression measurement. Other two meta-analyses [12,13]were published on this topic in the past 10 years and the most recent one was published in 2007. The meta-analysis [14] in 2007 reported a pooled relative risk estimated of 1.48 (95% CI, 1.29–1.69) for CHD, which focused on the cardiovascular outcomes and took pooled effect size of CHD from 16 studies published before 2005 as a secondary analysis. Furthermore, the review did not exclude those studies that participants were with CHD at the study baseline, which did not clearly enable reader to discern the depression as pre-morbid risk factor, and affected the magnitude of the true association. Potential publication bias was also not fully explored in this review. Since the publication of the last review on this topic, many more prospective studies have emerged, which allowed to perform more detailed analysis for new insights and obtain more powerful evidence on the association between depression and CHD risk. Given the limitations of previous reviews and the higher level of evidence from prospective cohort studies, along with the additional recent studies, it was necessary for us to assess the association between depression and the risk of CHD by conducting an updated meta-analysis based on prospective cohort studies.

## Methods

### Search strategy

We performed this systematic review in accordance with the Meta-analysis of Observational Studies in Epidemiology (MOOSE) guidelines [15]. A literature search of PubMed, Embase and Web of Science from their inception to April 2014 for prospective cohort studies describing the association between depression status (defined by self-reported scales, clinician/physician diagnosis, or structured clinical diagnostic interview) and the risk of CHD was conducted. We used the following search terms “depression,” “depressive symptoms,” “depression disorder,” and “coronary heart disease” or “ischemic heart disease” or “myocardial infarction” [MeSH] or “cardiovascular diseases” [MeSH] combined with “cohort studies” “follow-up studies” “prospective studies” “longitudinal studies”. In addition, the reference lists of all identified relevant publications and relevant reviews were reviewed. Only articles published in English were considered.

### Selection criteria

Studies were included in the meta-analysis if they met the following criteria: (1) the study was a population-based or community-based prospective cohort study design; (2) the exposure of interest was depression symptoms; (3) the outcome of interest was CHD or MI; (4) participants were free of CHD at study entry; (5) the study reported the risk estimates with 95% confidence interval (CI) for the association between depression and CHD. Studies were excluded if (1) the study had a retrospective design; (2) the estimates were presented without standard errors or other information that allowed calculation of standard errors; (3) no confounders were adjusted for. In the case of multiple publications from the same study, only the most recent paper or article with a longer follow-up was included.

### Data extraction

We extracted the following information from each retrieved study: name of the first author, year of publication, study location, characteristics of study population at baseline, duration of follow-up, definition and measurement of exposure, outcomes, number of cases, size of cohort, adjusted relative risk (RR) with 95% CI, and covariates that were adjusted in the multivariable analysis. Data extraction was conducted independently by two authors (Y.G. and Y.H.G). Interobserver agreement was assessed using Cohen kappa (κ), and any differences were resolved by discussion with the third author (Z.X.L.).

### Quality assessment

To determine the validity of included studies, two reviewers (Y.G. and Y.H.G.) independently performed the quality assessment by using the Newcastle-Ottawa Scale [16], which is a validated scale for non-randomized studies in meta-analyses [17]. The Newcastle-Ottawa Scale is a nine-point scale that allocates points based on the selection process of cohorts (0–4 points), the comparability of cohorts (0–2 points), and the identification of the exposure and the outcomes of study participants (0–3 points). We assigned scores of 0–3, 4–6, and 7–9 for low, moderate, and high quality of studies, respectively. Each study was rated independently by two authors (Y.G. and Y.H.G.); ratings were reported in Additional file 1: Table S1.

### Statistical analysis

In this meta-analysis, the RR was used as a common measure of the association between depression and the risk of CHD, and the hazard ratios (HRs) was considered equivalent to RRs. If articles provided RRs for women and men separately, we pooled both risk estimates to obtain one overall estimates for the primary analysis. If studies reported fatal or nonfatal outcomes, the two outcomes were considered as two independent reports respectively for all analysis. To be consistent across studies, we used binary variables (yes/no) for depression and CHD. We did not include studies using depressive scale as a continuous variable because the risk estimates were not comparable with those using categorized depression measures.

The heterogeneity among studies was estimated by the Cochran *Q* test and *I*^*2*^ statistic. *P* < 0.10 was considered indicative of statistically significant heterogeneity. Statistical heterogeneity among studies was evaluated using the *I*^*2*^ statistic, where values of 25%, 50% and 75% represent cut-off points for low, moderate and high degrees of heterogeneity, respectively [18]. When appropriate, we used a fixed-effects model or random-effects model. The RRs were pooled using the fixed-effects model if no heterogeneity was detected, or the random-effects model was used otherwise [19].

We conducted stratified analyses and sensitive analyses to evaluate the influences of study and population characteristic on study results. We used the Begg test [20], the Egger test [21] and visual inspection of a funnel plot to assess the publication bias. The Duval and Tweedie nonparametric trim-and-fill method [22] was performed to further assess the potential publication bias. All analyses were performed with STATA statistical software version 11.0 (STATA Corp, College Station, TX, USA). All tests were two sided with a significance level of 0.05.

## Results

### Literature search and study selection

**Figure 1:**
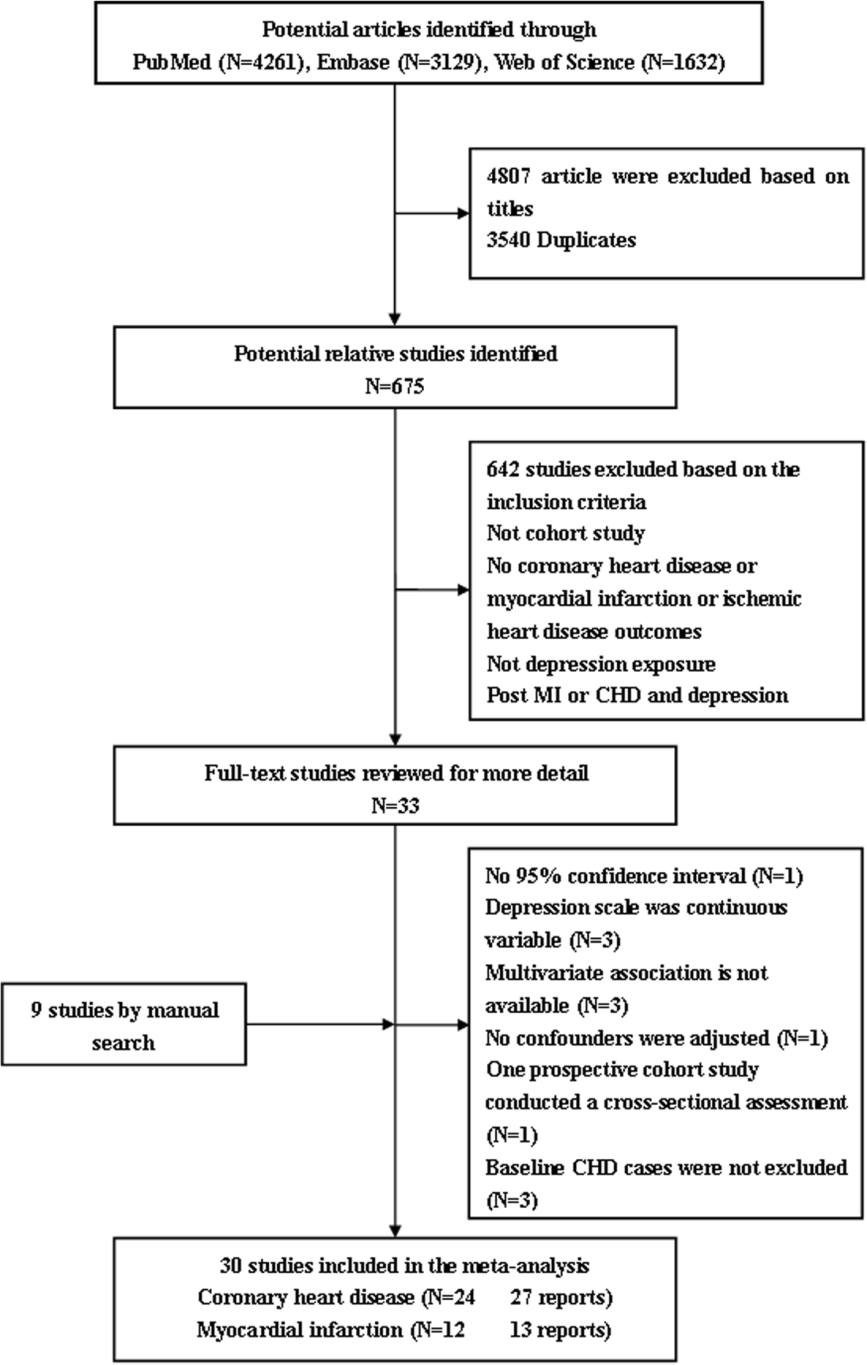
**Flow chart of study selection.**

Initially, 9022 articles from the PubMed, Embase, and Web of Science were identified. The majority were excluded after the first screening of titles or abstracts, because they were duplicates, reviews, case–control studies, cross-sectional studies, or not relevant to our analysis. After assessing full texts for detailed evaluation, 30 studies [23-52] met the inclusion criteria and were included in the meta-analysis. A flow chart showing the study selection was presented in Figure 1. Interobserver agreement (κ) was 0.959, which indicated a very outstanding concordance between raters for article inclusion decisions [53]. The quality of studies was generally good, with results of study quality assessment yielded a score of 6 or above for all included studies, with an average score of 7.8.

### Study characteristics

Additional file 1: Table S1 showed the main characteristics of 30 prospective cohort studies included that were published between 1993 and 2014. With regard to study location, fifteen studies were conducted in the United States, twelve in European countries (one of the twelve studies was a multi-country study), one each in Hong Kong, Taiwan, and Canada. The study samples ranged from 660 to 345,949, with a total of 893,850, and the follow-up durations ranged from 2 to 37 years. The number of CHD cases diagnosed in the primary studies ranged from 45 to 11,659, with 59,062 reported CHD outcomes (two studies [24,54] did not report the number of CHD cases). Twenty-three studies reported results for both men and women, six reported results for men and women separately (The result of one study is only available for men [33]), five studies reported results for men only, and two studies reported results for women only. One study [44] reported results by time interval: 0 to 5 year and 5 to 10 year. Three studies [23,29,46] reported the outcome of fatal and nonfatal CHD simultaneously. The assessment of depression varied across studies, twenty-two studies administered a validated questionnaire of depression symptoms, in which nine different self-reported symptom scales were represented in Additional file 1: Table S1. Twelve of twenty-two studies used the Center for Epidemiology Studies Depression scale (CESD). Of the 30 studies, 24 reported CHD outcomes and 12 reported MI outcomes. The proportion of major depression or scoring above the cutoff of self-rating instruments ranged from 1.3 to 50.6% (average 17.5%). The prevalence rate of depression symptoms was much higher than clinical diagnosis. Outcome ascertainment was from a variety of sources, such as medical records, self-report, register database, National Death Index, clinical diagnoses, and death certificates. The major adjustment confounding factors included age, sex, smoking, alcohol intake, physical activity, body mass index (BMI), diabetes, hypertension and cholesterol.

### Association between depression and the risk of all CHD events

**Figure 2:**
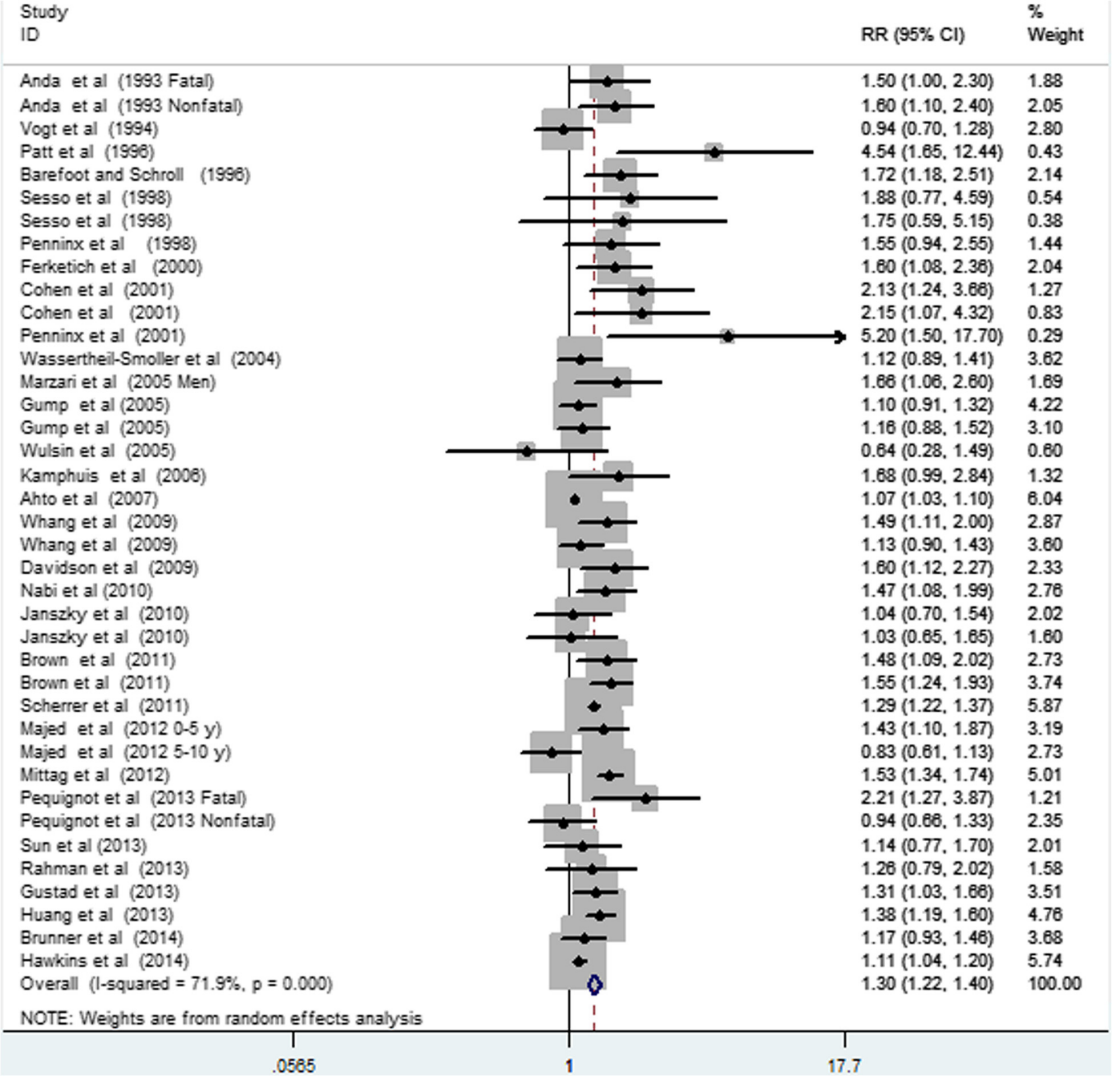
**Forest plot of depression and the risk of coronary heart disease.**

Thirty studies with 39 reports were included in the analysis of depression and CHD risk. The results from random-effects meta-analysis of depression and the risk of CHD were shown in Figure 2. Of the 39 reports, 21 showed a significantly positive relationship between depression and the risk of CHD, while the other reports did not. The pooled RR of CHD for depression was 1.30 (95%CI, 1.22–1.40). Substantial heterogeneity was observed *(P* < 0.001; *I*^*2*^ = 71.9%).

### Association of depression with the risk of MI

**Figure 3:**
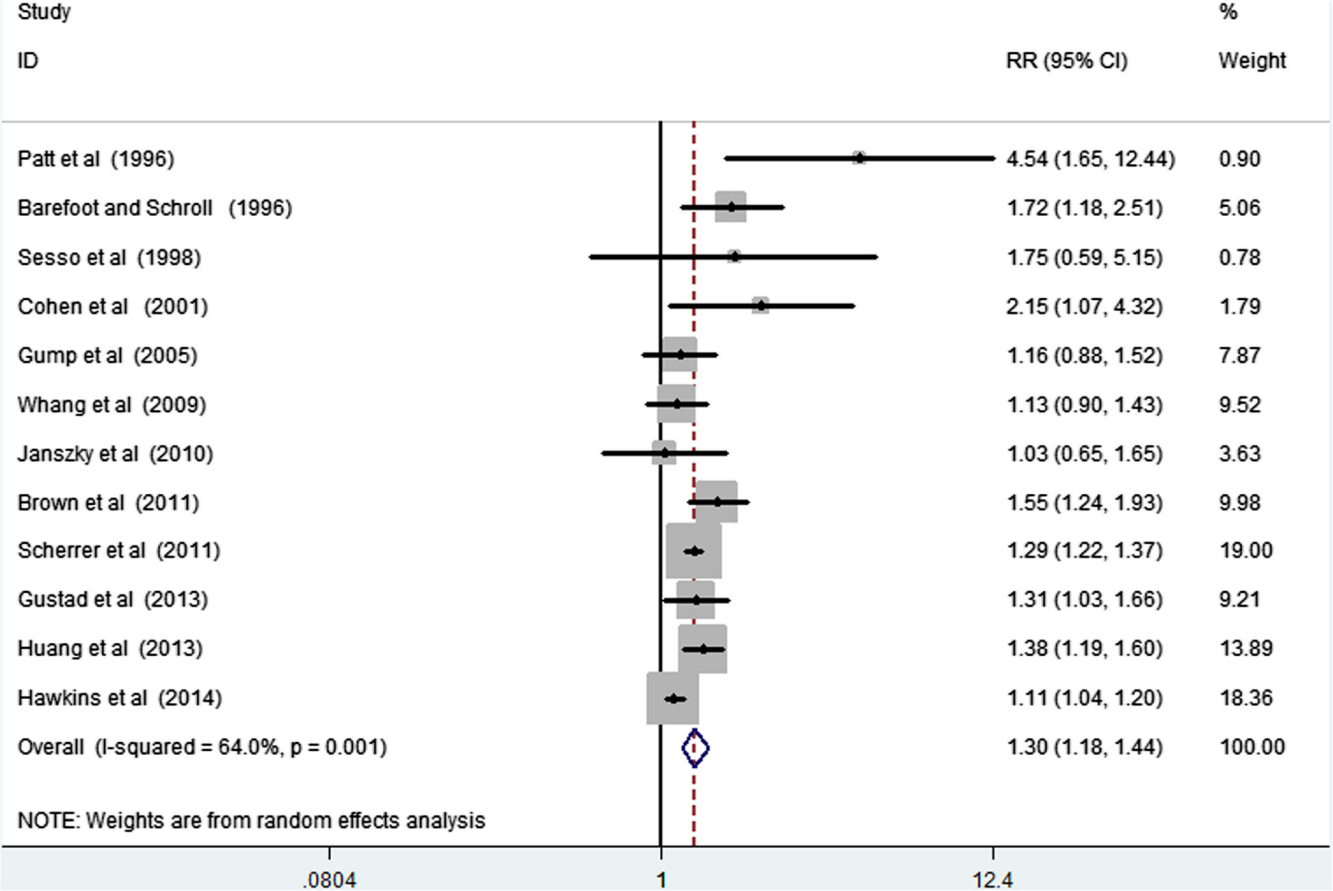
**Forest plot of depression and the risk of myocardial infarction.**

The results from random-effects meta-analysis of depression and the risk of MI were shown in Figure 3. Of the 12 studies, 8 showed a significant positive association between depression and MI risk, and 4 suggested no statistically significant association of interest. The pooled RR was 1.30 (95% CI, 1.18–1.44), and there was a moderate to high heterogeneity (*P* = 0.001; *I*^*2*^ = 64%). Visual inspection of the Begg funnel plot did not identify substantial asymmetry. Neither the Begg test nor the Egger test for publication bias reached significance (*P* > 0.05 for both tests).

Of note, one study [55] was excluded because depressive symptoms score was used as a continuous variable. We added the RR from the study to the main analysis of MI, and the pooled RR was 1.26 (95 CI%, 1.16 to 1.37). Thus, our results would not change even if the study was included.

### Subgroup analyses

Table 1, Additional file 1: Table S2 and S3 showed the results from subgroup analyses examining the stability of the primary results and exploring the resource of heterogeneity. The associations between depression and the risk of CHD and MI were similar in most subgroup analyses, which were stratified by sex, mean age at baseline, publication year (before 2005 vs. after 2005), duration of follow-up, study location, type of depression measure, and whether smoking, BMI, diabetes, hypertension, physical activity, cholesterol or socioeconomic status were controlled or not in models. However, moderate to high heterogeneities were observed in most subgroups. The increased CHD risk was more evident in the groups of less than 15 years follow-up and adjusted for hypertension. No significant between-group difference was found for other variable (see Additional file 1: Table S2 and S3).

**Table 1:** **Subgroup analyses of relative risk of coronary heart disease**

	**No of reports**	**Relative risk**	**(95% CI)**	**I** ^**2**^	**P for heterogeneity**
**Coronary heart disease**					
Primary analysis	27*	1.31	1.19 to 1.45	70.50%	<0.001
**Subgroup analyses for coronary heart disease**					
Sex					
Men	13	1.38	1.17 to 1.61	74.60%	<0.001
Women	8	1.17	1.01 to 1.36	55.50%	0.028
Combined	13	1.36	1.17 to 1.57	56.50%	0.006
Mean age at baseline, y					
≥65	13	1.35	1.14 to 1.59	81.20%	<0.001
<65	21	1.3	1.17 to 1.44	61.40%	<0.001
Publication year					
Before 2005	15	1.47	1.23 to 1.75	62.30%	0.001
2005-2014	19	1.24	1.13 to 1.37	74.30%	<0.001
Duration of follow-up					
≥15 years	4	1.09	0.96 to 1.23	0.00%	0.714
<15 years	30	1.36	1.24 to 1.49	74.80%	<0.001
Study location					
Unite States, Canada	21	1.39	1.24 to 1.56	53.20%	0.002
Europe	11	1.18	1.06 to 1.32	69.70%	<0.001
Asia	2	1.14	0.77 to 1.70	81.20%	0.021
Type of depression measurement					
Self-reported scales	29	1.33	1.22 to 1.46	74.30%	<0.001
Clinical diagnosis	4	1.2	0.82 to 1.74	60.00%	0.058
Combined	1	1.12	0.89 to 1.41	NA	NA

Abbreviations: NA, not applicable.

*Five articles provided RRs for women and men separately, we pooled both risk estimates to obtain one overall estimates for the primary analysis; therefore, there are 27 reports in the primary analysis.

### Sensitivity analyses

Sensitivity analyses were performed to detect potential sources of heterogeneity in the association between depression and the risk of CHD, and to examine the influence of various exclusion criteria on the combined risk estimate. Exclusion of 3 studies that their outcomes were CHD plus MI showed a greater risk 1.35 (95% CI, 1.22–1.50), and statistical heterogeneity was forcefully attenuated (*P* =0.001, *I*^*2*^ = 52.8%). Exclusion of the study by Cohen et al. that enrolled patients with hypertension yielded a pooled RR of 1.29 (95%CI, 1.17-1.43; *P* < 0.001). We excluded any single study in turn and pooled the results of the remaining studies. The overall combined RR did not change substantially, with a range from 1.29 (95% CI, 1.17 to 1.42; *P* <0.001) to 1.34 (95%CI, 1.21 to 1.48; *P* <0.001).

Two studies [56,57] were excluded because depressive symptoms score was used as a continuous variable rather than a binary variable. We added the RRs from the two studies to the main analysis of CHD, and the results did not change substantially (RR, 1.25; 95% CI, 1.16 to 1.34).

### Publication bias

**Figure 4:**
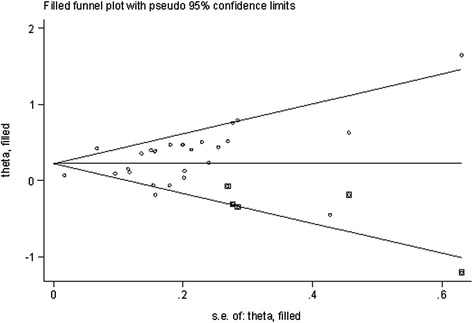
**Filled funnel of relative risk of studies that investigated the association between depression and the risk of coronary heart disease.**

Visual inspection of the Begg funnel plot did not reveal any asymmetry. The Begg test was not significant (*z* = 1.13; *P* = 0.26), which identified no evidence of substantial publication bias, but the Egger test did not (*P* = 0.059). A sensitivity analysis using the trim-and-fill method was performed with 5 imputed studies, which produced a symmetrical funnel plot (see Figure. 4). The corrected RR using the trim-and-fill method was 1.25 (95% CI, 1.14–1.38; *P* < 0.001). Correction for potential publication bias therefore did not alter the combined risk estimate substantially.

## Discussion

The results of our meta-analysis of 30 prospective cohort studies with 40 independent reports suggest that depression is associated with a significantly increased risk of CHD and MI. Participants with depression, compared with those free of it, experienced a significant increased risk of 30% for CHD and MI. Furthermore, the association remained significant in the groups adjusted for potential confounders, such as lifestyle factors and socio-demographic factors.

### Comparison with previous studies

The present meta-analysis showed that the pooled adjusted RRs were 1.30 (95% CI, 1.22–1.40), 1.30 (95% CI, 1.18–1.44) for CHD and MI, respectively, which were much lower than those from a previous meta-analysis published 2007 for CHD (RR, 1.48; 95% CI, 1.29–1.69) and for MI (RR, 1.60; 95% CI, 1.34–1.92). Our meta-analysis included 19 new prospective cohort studies with larger sample size and many more cases, which significantly enhanced statistical power to detect the potential associations of depression with CHD and MI. In addition, we also fully explored the potential publication bias. Although potential publication bias may exist, correction for this bias using trim-and-fill method remained statistically significant. More important, compared with the previous meta-analysis in 2007, the associations differed between populations of different ethnic backgrounds was investigated. We found that the association trended to be stronger for participants from the United States than for European participants. There was null statistically significant risk of CHD in Hong Kong, which might result from the limited number of included studies (One study with two reports comprising 62,839 participants). Given that the studies included in our meta-analysis were conducted in affluent countries (areas) in North America, Western Europe, and Hong Kong, the results should not be extended to developing countries. In order to make the finding generalize to other populations, more studies conducted in other populations from Asia, Africa and South America are warranted.

Our subgroups analyses identified two important and valuable findings. A major finding was that depression increased the risk of CHD in the group of less than 15 years follow-up, but did not have statistically significant association for equal to or more than 15 years follow-up, which was a very interesting phenomenon. One possible explanation to this finding was that most studies have usually measured depression only one time at the beginning of the study, and have assessed the outcomes at the end of the follow-up. It was well known that depression was treatable and depressive individuals could recover during the follow-up. The longer the follow-up duration, the more people could recover, which might weaken the association between depression and CHD risk. In turn, the finding confirmed that the depression was associated with CHD risk.

Another finding was that depression could be an independent risk factor for CHD. In our subgroup analyses, studies adjusted for smoking, BMI, hypertension, diabetes, physical activity, and socioeconomic status did not influence the result of positive association, which suggested that adequate adjustment was not definite to weaken the pooled effect estimate and conclusion [58].

In the subgroup analysis type of depression measurement, it was notable that the association was much stronger in these studies that identified depression using self-reported scales rather than structured clinical diagnostic interviews or clinical diagnosis. One possible interpretation was that the estimates of depression may differ depending on the use of dimensionally versus categorically based depression assessment tools [59]. Structured psychiatric interviews would definitely exclude individuals with subsyndromal depressive symptoms from case status. Conversely, utilization of self-reported symptom scales would allow the inclusion of a lot of people with clinically significant depressive symptoms who failed to meet formal criteria for diagnostic and statistical manual of mental disorders (DSM) diagnosis, but a large body of evidence showed that subsyndromal depressive symptoms, like clinical syndromes, were significantly associated with adverse functional outcomes, disability, morbidity and mortality [60]. Therefore, inclusion of people with subsyndromal depression in the reference category may weaken the risk estimates of studies with categorically based depression definition. More important, there were quite clear differences present that go opposite to a dose–response association between depression and CHD. The interesting finding deserves attention from related researchers. More studies investigating the association between depression and CHD risk based on the use frequency of self-report instruments and interviews over time are needed, which will help to explore the dose–response relationship with them. Specially, in our meta-analysis, a majority of studies defined depression status by self-reported symptom scales, thus our findings much more trended to generalize to the populations who had depression symptoms.

On the basis of our research findings, it was expected that treatment with antidepressants would reduce the risk of development of CHD. Conversely, some observational studies [40,61] suggested treatment with antidepressants was associated with increasing risk of CHD. However, the results should be interpreted cautiously because medication use could be a marker of depression severity [62], evidence from a meta-analysis of clinical trials showed that treatment with antidepressants was beneficial for CHD [63]. Thus, patients with antidepressants medication use were more likely to have severe depressive symptoms, and the increased risk of CHD may be attributable to depression severity rather than the antidepressant medication use.

Interestingly, although the evidence from observational studies on the association between depression and the risk of CHD seemed to be robust, the evidence from randomized controlled trials (RCTs) did not find an effect of the intervention [64,65]. We noted that evidence from RCTs had suggested that the treatment response and medical prognosis of different patient subgroups appear different [66]. For example, among post–acute coronary syndrome patients, depression, particularly if mild, might be monitored for remission without treatment. On the contrary, persistent depression, particularly if treatment recalcitrant, should be treated, taking into considering the link to adverse post-acute coronary syndrome outcomes [66]. In addition, studies suggested that gender might play a different role in affecting the medical outcomes from psychotherapy [67,68]. Therefore, more research is needed to further investigate the prognosis of subgroups based on depression severity and/or onset and gender, which would help to reveal the relationship between depression and CHD.

### Study strengths and limitations

Our review is very valuable and crucial though it is an updated meta-analysis. Firstly, we not only included the prospective cohort studies, also included 19 more new studies and 759,922 more new participants than the previous reviews, which provided stronger and more sufficient evidence. Secondly, on the basis of our subgroup analysis, an important methodological finding was worth paying attention to. In cohort studies with longer follow-up, exposure (such as depression) might be mutative, but the exposure measurement frequency was often inadequate, which should arouse the investigator’s attention in the population-based observational epidemiological studies.

A few limitations of our meta-analysis should be acknowledged. Firstly, we observed robust and consistent associations across different subgroups via sensitivity analyses and subgroup analyses. Yet as a limitation, there was the evidence of heterogeneity across the studies used for the analysis of association between depression and the risk of CHD. The heterogeneity might result from the difference of participants’ characteristic, sample sizes, study designs, and diagnostic criteria of depression. Thus, the results of this meta-analysis should be interpreted cautiously. Secondly, the measurement of depression mostly used the self-reported symptom scales, which could cause the misclassification of exposure [9], and might underestimate the reported association. Thirdly, the publication bias were observed, however, we used trim-and-fill method to correct the bias, which did not obviously alter the positive association. Fourthly, the receipt and type of depression treatment were not taken into consideration, and many studies lacked information on depression treatment and antidepressant medication use. The role of depression treatment in modulating subsequent risk of CHD needs to be studied further.

### Suggestions for further studies

On the basis of our findings, we put forward some suggestions for future studies. Firstly, more studies are needed to assess depression in a repeated-measures design with diagnostic interview as well as self-report during the follow-up. Secondly, in order to achieve stronger research evidence, investigators should strive to improve the standardization of their depression measurement and outcome definitions [69]. Thirdly, we should inquire the variation of depression of participants at the end of the follow-up, and further accurately assess the association between depression and the risk of CHD. Finally, more interventional studies are needed to explore the underlying mechanisms and to determine the cause and effect relationships that link depression and CHD.

## Conclusion

In conclusion, our meta-analysis of prospective studies suggests that depression is probably an independent risk factor, which is associated with a 30% increase risk for CHD and MI. Given the high prevalence of depression in the general population and the heavy economic burden of CHD, it is greatly important for us to take depression account into the clinical prevention and treatment of CHD.

## Additional file

Additional file 1:
**Table S1.** Characteristics of studies included in the meta-analysis. **Table S2.** Subgroup analyses of relative risk of coronary heart disease. **Table S3.** Subgroup analyses of relative risk of myocardial infarction.

## Abbreviations

BMI: Body mass index
BDI: Beck Depression Inventory
CI: Confidential interval
CES-D: Center for Epidemiologic Studies Depression Scale
CHD: Coronary heart disease
CVD: Cardiovascular disease
CBVD: Cerebrovascular disease
DBP: Diastolic blood pressure
DIS: Diagnostic interview schedule
GDS: Geriatric Depression Scale
GHQ: General Health Questionnaire
GWB-D: General Well-Being Schedule-Depressed Mood
HR: Hazard ratio
IHD: Ischemic heart disease
MHI-5: 5-item Mental Health Index
MI: Myocardial infarction
MMPI: Minnesota Multiphasic Personality Inventory
NA: Not applicable
OBD: 40 items Obvious Depression Subscale
OR: Odds ratio
RR: Relative risk
SBP: Systolic blood pressure
SDS: Zung Self-Rating Depression Scale
SZS: Short Zung Depression Scale

## References

[1] Ford ES, Capewell S (2007). Coronary heart disease mortality among young adults in the U.S. from 1980 through 2002: concealed leveling of mortality rates. J Am Coll Cardiol.

[2] Roger VL, Go AS, Lloyd-Jones DM, Benjamin EJ, Berry JD, Borden WB, Bravata DM, Dai S, Ford ES, Fox CS, Fullerton HJ, Gillespie C, Hailpern SM, Heit JA, Howard VJ, Kissela BM, Kittner SJ, Lackland DT, Lichtman JH, Lisabeth LD, Makuc DM, Marcus GM, Marelli A, Matchar DB, Moy CS, Mozaffarian D, Mussolino ME, Nichol G, Paynter NP, Soliman EZ, Sorlie PD, Sotoodehnia N, Turan TN, Virani SS, Wong ND, Woo D, Turner MB, American Heart Association Statistics Committee and Stroke Statistics Subcommittee (2012). Executive summary: heart disease and stroke statistics–2012 update: a report from the American Heart Association. Circulation.

[3] Weintraub WS, Daniels SR, Burke LE, Franklin BA, Goff DC, Hayman LL, Lloyd-Jones D, Pandey DK, Sanchez EJ, Schram AP, Whitsel LP, American Heart Association Advocacy Coordinating Committee; Council on Cardiovascular Disease in the Young; Council on the Kidney in Cardiovascular Disease; Council on Epidemiology and Prevention; Council on Cardiovascular Nursing; Council on Arteriosclerosis; Thrombosis and Vascular Biology; Council on Clinical Cardiology, and Stroke Council (2011). Value of primordial and primary prevention for cardiovascular disease: a policy statement from the American Heart Association. Circulation.

[4] Perk J, De Backer G, Gohlke H, Graham I, Reiner Z, Verschuren M, Albus C, Benlian P, Boysen G, Cifkova R, Deaton C, Ebrahim S, Fisher M, Germano G, Hobbs R, Hoes A, Karadeniz S, Mezzani A, Prescott E, Ryden L, Scherer M, Syvänne M, op Reimer WJ S, Vrints C, Wood D, Zamorano JL, Zannad F, European Association for Cardiovascular Prevention & Rehabilitation (EACPR); ESC Committee for Practice Guidelines (CPG) (2012). European Guidelines on cardiovascular disease prevention in clinical practice (version 2012). The Fifth Joint Task Force of the European Society of Cardiology and Other Societies on Cardiovascular Disease Prevention in Clinical Practice (constituted by representatives of nine societies and by invited experts). Eur Heart J.

[5] Greenland P, Alpert JS, Beller GA, Benjamin EJ, Budoff MJ, Fayad ZA, Foster E, Hlatky MA, Hodgson JM, Kushner FG, Lauer MS, Shaw LJ, Smith SC Jr, Taylor AJ, Weintraub WS, Wenger NK, Jacobs AK, Smith SC Jr, Anderson JL, Albert N, Buller CE, Creager MA, Ettinger SM, Guyton RA, Halperin JL, Hochman JS, Kushner FG, Nishimura R, Ohman EM, Page RL et al: **2010 ACCF/AHA guideline for assessment of cardiovascular risk in asymptomatic adults: a report of the American College of Cardiology Foundation/American Heart Association Task Force on Practice Guidelines.** *J Am Coll Cardiol* 2010, **56**(25):e50–103. 10.1016/j.jacc.2010.09.001 21144964

[6] The World Health Organization.The World Health Report 2001: Mental Health: new understanding, new hope.http://www.who.int/whr/2001/en/.

[7] Bromet E, Andrade LH, Hwang I, Sampson NA, Alonso J, de Girolamo G, de Graaf R, Demyttenaere K, Hu C, Iwata N, Karam AN, Kaur J, Kostyuchenko S, Lépine JP, Levinson D, Matschinger H, Mora ME, Browne MO, Posada-Villa J, Viana MC, Williams DR, Kessler RC: **Cross-national epidemiology of DSM-IV major depressive episode.** *BMC Med* 2011, **9:**90. 10.1186/1741-7015-9-90 PMC3163615 21791035

[8] LA Murray CJL (1996). The Global Burden of Disease: A comprehensive assessment of mortality and disability from diseases, injuries and risk factors in 1990 and projected to 2020.

[9] Dong JY, Zhang YH, Tong J, Qin LQ (2012). Depression and risk of stroke: a meta-analysis of prospective studies. Stroke.

[10] Booth-Kewley S, Friedman HS (1987). Psychological predictors of heart disease: a quantitative review. Psychol Bull.

[11] Rugulies R (2002). Depression as a predictor for coronary heart disease. a review and meta-analysis. Am J Prev Med.

[12] Wulsin LR, Singal BM (2003). Do depressive symptoms increase the risk for the onset of coronary disease? A systematic quantitative review. Psychosom Med.

[13] Nicholson A, Kuper H, Hemingway H (2006). Depression as an aetiologic and prognostic factor in coronary heart disease: a meta-analysis of 6362 events among 146 538 participants in 54 observational studies. Eur Heart J.

[14] Van der Kooy K, van Hout H, Marwijk H, Marten H, Stehouwer C, Beekman A (2007). Depression and the risk for cardiovascular diseases: systematic review and meta analysis. Int J Geriatr Psychiatry.

[15] Stroup DF, Berlin JA, Morton SC, Olkin I, Williamson GD, Rennie D, Moher D, Becker BJ, Sipe TA, Thacker SB (2000). Meta-analysis of observational studies in epidemiology: a proposal for reporting. Meta-analysis Of Observational Studies in Epidemiology (MOOSE) group. JAMA.

[16] Wells G, Shea B, O'Connell D, Peterson J, Welch V, Losos M: **The Newcastle-OttawaScale(NOS) for assessingthe quality of nonrandomized studies in meta-analyses.** Available at http://www.ohri.ca/programs/clinical_epidemiology/oxford.asp.

[17] Rong Y, Chen L, Zhu T, Song Y, Yu M, Shan Z, Sands A, Hu FB, Liu L: **Egg consumption and risk of coronary heart disease and stroke: dose–response meta-analysis of prospective cohort studies**. *BMJ (Clinical research ed)* 2013, **346**:e8539 10.1136/bmj.e8539 PMC3538567 23295181

[18] Higgins JP (2008). Commentary: Heterogeneity in meta-analysis should be expected and appropriately quantified. Int J Epidemiol.

[19] Lau J, Ioannidis JP, Schmid CH (1997). Quantitative synthesis in systematic reviews. Ann Intern Med.

[20] Begg CB, Mazumdar M (1994). Operating characteristics of a rank correlation test for publication bias. Biometrics.

[21] Egger M, Davey Smith G, Schneider M, Minder C (1997). Bias in meta-analysis detected by a simple, graphical test. BMJ (Clinical research ed).

[22] Duval S, Tweedie R (2000). Trim and fill: A simple funnel-plot-based method of testing and adjusting for publication bias in meta-analysis. Biometrics.

[23] Anda R, Williamson D, Jones D, Macera C, Eaker E, Glassman A, Marks J: **Depressed affect, hopelessness, and the risk of ischemic heart disease in a cohort of U.S. adults.** *Epidemiology (Cambridge, Mass)* 1993, **4**(4):285–294 10.1097/00001648-199307000-00003 8347738

[24] Vogt T, Pope C, Mullooly J, Hollis J (1994). Mental health status as a predictor of morbidity and mortality: a 15-year follow-up of members of a health maintenance organization. Am J Public Health.

[25] Pratt LA, Ford DE, Crum RM, Armenian HK, Gallo JJ, Eaton WW (1996). Depression, psychotropic medication, and risk of myocardial infarction. Prospective data from the Baltimore ECA follow-up. Circulation.

[26] Barefoot JC, Schroll M (1996). Symptoms of depression, acute myocardial infarction, and total mortality in a community sample. Circulation.

[27] Penninx BW, Guralnik JM, de Leon CF M, Pahor M, Visser M, Corti MC, Wallace RB (1998). Cardiovascular events and mortality in newly and chronically depressed persons > 70 years of age. Am J Cardiol.

[28] Sesso HD, Kawachi I, Vokonas PS, Sparrow D (1998). Depression and the risk of coronary heart disease in the Normative Aging Study. Am J Cardiol.

[29] Ferketich AK, Schwartzbaum JA, Frid DJ, Moeschberger ML (2000). Depression as an antecedent to heart disease among women and men in the NHANES I study. National Health and Nutrition Examination Survey. Arch Intern Med.

[30] Penninx BW, Beekman AT, Honig A, Deeg DJ, Schoevers RA, van Eijk JT, van Tilburg W (2001). Depression and cardiac mortality: results from a community-based longitudinal study. Arch Gen Psychiatry.

[31] Cohen HW, Madhavan S, Alderman MH (2001). History of treatment for depression: risk factor for myocardial infarction in hypertensive patients. Psychosom Med.

[32] Wassertheil-Smoller S, Shumaker S, Ockene J, Talavera GA, Greenland P, Cochrane B, Robbins J, Aragaki A, Dunbar-Jacob J (2004). Depression and cardiovascular sequelae in postmenopausal women. The Women's Health Initiative (WHI). Arch Intern Med.

[33] Marzari C, Maggi S, Manzato E, Destro C, Noale M, Bianchi D, Minicuci N, Farchi G, Baldereschi M, Di Carlo A, Crepaldi G: **Depressive symptoms and development of coronary heart disease events: the Italian longitudinal study on aging.** *J Gerontol A Biol Sci Med Sci* 2005, **60**(1):85–92. 10.1093/gerona/60.1.85 15741288

[34] Gump BB, Matthews KA, Eberly LE, Chang YF (2005). Depressive symptoms and mortality in men: results from the Multiple Risk Factor Intervention Trial. Stroke.

[35] Wulsin LR, Evans JC, Vasan RS, Murabito JM, Kelly-Hayes M, Benjamin EJ (2005). Depressive symptoms, coronary heart disease, and overall mortality in the Framingham Heart Study. Psychosom Med.

[36] Kamphuis MH, Kalmijn S, Tijhuis MA, Geerlings MI, Giampaoli S, Nissinen A, Grobbee DE, Kromhout D (2006). Depressive symptoms as risk factor of cardiovascular mortality in older European men: the Finland, Italy and Netherlands Elderly (FINE) study. Eur J Cardiovasc Prev Rehabil.

[37] Ahto M, Isoaho R, Puolijoki H, Vahlberg T, Kivela SL (2007). Stronger symptoms of depression predict high coronary heart disease mortality in older men and women. Int J Geriatr Psychiatry.

[38] Whang W, Kubzansky LD, Kawachi I, Rexrode KM, Kroenke CH, Glynn RJ, Garan H, Albert CM (2009). Depression and risk of sudden cardiac death and coronary heart disease in women: results from the Nurses' Health Study. J Am Coll Cardiol.

[39] Davidson KW, Schwartz JE, Kirkland SA, Mostofsky E, Fink D, Guernsey D, Shimbo D (2009). Relation of inflammation to depression and incident coronary heart disease (from the Canadian Nova Scotia Health Survey [NSHS95] Prospective Population Study). Am J Cardiol.

[40] Nabi H, Kivimaki M, Suominen S, Koskenvuo M, Singh-Manoux A, Vahtera J (2010). Does depression predict coronary heart disease and cerebrovascular disease equally well? The Health and Social Support Prospective Cohort Study. Int J Epidemiol.

[41] Janszky I, Ahnve S, Lundberg I, Hemmingsson T (2010). Early-onset depression, anxiety, and risk of subsequent coronary heart disease: 37-year follow-up of 49,321 young Swedish men. J Am Coll Cardiol.

[42] Brown JM, Stewart JC, Stump TE, Callahan CM (2011). Risk of coronary heart disease events over 15 years among older adults with depressive symptoms. Am J Geriatr Psychiatry.

[43] Scherrer JF, Garfield LD, Chrusciel T, Hauptman PJ, Carney RM, Freedland KE, Owen R, True WR, Lustman PJ (2011). Increased risk of myocardial infarction in depressed patients with type 2 diabetes. Diabetes Care.

[44] Majed B, Arveiler D, Bingham A, Ferrieres J, Ruidavets JB, Montaye M, Appleton K, Haas B, Kee F, Amouyel P, Ducimetiere P, Empana JP: **Depressive symptoms, a time-dependent risk factor for coronary heart disease and stroke in middle-aged men: the PRIME Study.** *Stroke* 2012, **43**(7):1761–1767. 10.1161/STROKEAHA.111.645366 22517599

[45] Mittag O, Meyer T (2012). The association of depressive symptoms and ischemic heart disease in older adults is not moderated by gender, marital status or education. Int J Public Health.

[46] Pequignot R, Tzourio C, Peres K, Ancellin ML, Perier MC, Ducimetiere P, Empana JP (2013). Depressive symptoms, antidepressants and disability and future coronary heart disease and stroke events in older adults: the Three City Study. Eur J Epidemiol.

[47] Sun WJ, Xu L, Chan WM, Lam TH, Schooling CM (2013). Are depressive symptoms associated with cardiovascular mortality among older Chinese: a cohort study of 64,000 people in Hong Kong?. Am J Geriatr Psychiatry.

[48] Gustad LT, Laugsand LE, Janszky I, Dalen H, Bjerkeset O: **Symptoms of anxiety and depression and risk of acute myocardial infarction: the HUNT 2 study.** *Eur Heart J*, 2014, **35**(21):1394–1403. 10.1093/eurheartj/eht387 PMC4043317 24057077

[49] Huang CJ, Hsieh MH, Hou WH, Liu JC, Jeng C, Tsai PS (2013). Depression, antidepressants, and the risk of coronary heart disease: a population-based cohort study. Int J Cardiol.

[50] Rahman I, Humphreys K, Bennet AM, Ingelsson E, Pedersen NL, Magnusson PK (2013). Clinical depression, antidepressant use and risk of future cardiovascular disease. Eur J Epidemiol.

[51] Brunner EJ, Shipley MJ, Britton AR, Stansfeld SA, Heuschmann PU, Rudd AG, Wolfe CD, Singh-Manoux A, Kivimaki M (2014). Depressive disorder, coronary heart disease, and stroke: dose–response and reverse causation effects in the Whitehall II cohort study. European journal of preventive cardiology.

[52] Hawkins MA, Callahan CM, Stump TE, Stewart JC (2014). Depressive symptom clusters as predictors of incident coronary artery disease: a 15-year prospective study. Psychosom Med.

[53] Altman DG (1999). Practical Statistics for Medical Research.

[54] Sun WJ, Xu L, Chan WM, Lam TH, Schooling CM: **Are Depressive Symptoms Associated With Cardiovascular Mortality Among Older Chinese: A Cohort Study of 64,000 People in Hong Kong?** *Am J Geriatr Psychiatry* 2013. 10.1016/j.jagp.2013.01.048 23567371

[55] Ika K, Suzuki E, Mitsuhashi T, Takao S, Doi H (2013). Shift work and diabetes mellitus among male workers in Japan: does the intensity of shift work matter?. Acta Med Okayama.

[56] Tranmer J (2013). Shift work and risk for type 2 diabetes. Can Nurse.

[57] Young J, Waclawski E, Young JA, Spencer J (2013). Control of type 1 diabetes mellitus and shift work. Occup Med (Lond).

[58] Meng L, Chen D, Yang Y, Zheng Y, Hui R (2012). Depression increases the risk of hypertension incidence: a meta-analysis of prospective cohort studies. J Hypertens.

[59] Kessler RC (2002). The categorical versus dimensional assessment controversy in the sociology of mental illness. J Health Soc Behav.

[60] Meeks TW, Vahia IV, Lavretsky H, Kulkarni G, Jeste DV (2011). A tune in "a minor" can "b major": a review of epidemiology, illness course, and public health implications of subthreshold depression in older adults. J Affect Disord.

[61] Hippisley-Cox J, Pringle M, Hammersley V, Crown N, Wynn A, Meal A, Coupland C (2001). Antidepressants as risk factor for ischaemic heart disease: case–control study in primary care. BMJ (Clinical research ed).

[62] Pan A, Sun Q, Okereke OI, Rexrode KM, Hu FB (2011). Depression and risk of stroke morbidity and mortality: a meta-analysis and systematic review. JAMA.

[63] Pizzi C, Rutjes AW, Costa GM, Fontana F, Mezzetti A, Manzoli L (2011). Meta-analysis of selective serotonin reuptake inhibitors in patients with depression and coronary heart disease. Am J Cardiol.

[64] Glassman AH, O'Connor CM, Califf RM, Swedberg K, Schwartz P, Bigger JT Jr, Krishnan KR, van Zyl LT, Swenson JR, Finkel MS, Landau C, Shapiro PA, Pepine CJ, Mardekian J, Harrison WM, Barton D, Mclvor M: **Sertraline treatment of major depression in patients with acute MI or unstable angina.** *JAMA* 2002, **288**(6):701–709. 10.1001/jama.288.6.701 12169073

[65] Berkman LF, Blumenthal J, Burg M, Carney RM, Catellier D, Cowan MJ, Czajkowski SM, DeBusk R, Hosking J, Jaffe A, Kaufmann PG, Mitchell P, Norman J, Powell LH, Raczynski JM, Schneiderman N: **Effects of treating depression and low perceived social support on clinical events after myocardial infarction: the Enhancing Recovery in Coronary Heart Disease Patients (ENRICHD) Randomized Trial.** *JAMA* 2003, **289**(23):3106–3116. 10.1001/jama.289.23.3106 12813116

[66] Ramamurthy G, Trejo E, Faraone SV: Depression treatment in patients with coronary artery disease: a systematic review. *The primary care companion to CNS disorders*. 2013, **15**(5). 10.4088/PCC.13r01509 PMC3907329 24511449

[67] Denollet J, Martens EJ, Nyklicek I, Conraads VM, de Gelder B: **Clinical events in coronary patients who report low distress: adverse effect of repressive coping.** *Health Psychol* 2008, **27**(3):302–308. 10.1037/0278-6133.27.3.302 18624593

[68] Schneiderman N, Saab PG, Catellier DJ, Powell LH, DeBusk RF, Williams RB, Carney RM, Raczynski JM, Cowan MJ, Berkman LF, Kaufmann PG: **Psychosocial treatment within sex by ethnicity subgroups in the Enhancing Recovery in Coronary Heart Disease clinical trial.** *Psychosom Med* 2004, **66**(4):475–483. 10.1097/01.psy.0000133217.96180.e8 15272091

[69] Frasure-Smith N, Lesperance F (2005). Reflections on depression as a cardiac risk factor. Psychosom Med.

