# Supplementary material for: Depression and the risk of coronary heart disease: a meta-analysis of prospective cohort studies
Source: BMC Psychiatry. 2014 Dec 24;14:371. doi: 10.1186/s12888-014-0371-z (PMC4336481; doi:10.1186/s12888-014-0371-z)
Supplement: Additional file 1: — Table S1. Characteristics of studies included in the meta-analysis. Table S2. Subgroup analyses of relative risk of coronary heart disease. Table S3. Subgroup analyses of relative risk of myocardial infarction. [file 12888_2014_371_MOESM1_ESM.docx]

**Supplemental material**

**Supplemental Table 1.Characteristics of studies included in the meta-analysis**

| **Sources** | **Population** | **Duration (years)** | **Depression assessment** | **NO of cases** | **Outcomes** | **Outcomes**  **measures** | **Adjustment for confounders** | **Quality assessment** | **Depression at baseline (%)** |
| --- | --- | --- | --- | --- | --- | --- | --- | --- | --- |
| Anda et al 1993 (Unite States) | 2832 men and women aged 45-77 mean=57.5 | 12.4 | GWB | 394 | IHD | Medical record and official death certificates and NDI | Age, gender, race, marital status, education, alcohol use, smoking, lipids, blood pressure, exercise, BMI | 9 | 11.1 |
| Vogt et al 1994 (Unite States) | 2573 men and women aged ≥18 mean<65 | 15 | Depressive Index | NA | IHD | Death index and vital records | Age, sex, socioeconomic status, length of health plan membership, subjective health status, smoking | 7 | NA |
| Patt et al 1996 (Unite States) | 1551 men and women aged≥18 mean<65 | 13 | DIS | 64 | MI | Self-report | Age; sex; marital status; blood pressure | 7 | 4.7 |
| Barefoot and Schroll 1996 (Denmark) | 730 men and women aged mean=77 | 26.2 | MMPI subscale OBD | 14 | MI | Medical records and Death certificates and autopsy reports | Age, sex | 8 | 18.1 |
| Penninx et al 1998 (Unite States) | 3701 men and women aged 70-103 mean=78.3 | 4 | CES-D | 537 | CHD | Medical records and death certificates | Age, smoking, alcohol, BMI, blood pressure, history of stroke, diabetes, cancer, physical disability | 7 | 12.9 |
| Sesso et al 1998 (Unite States) | 1305 men aged 40-90 mean=61.8(8.3） | 7 | MMPI-2 DEP | 50 | CHD,MI | Clinical diagnosis or autopsy and death certificates | Age, smoking, SBP, DBP, BMI, family history of CHD, alcohol | 7 | 24.1 |
| Ferketich et al 2000 (Unite States) | 7893 men and women aged≥30 men mean=55.9(14.4) women mean=53.7(13.9) | 10 | CES-D | 640 | CHD | Medical records and death certificates | Poverty index and (poverty index)2 smoking, hypertension, diabetes, BMI | 8 | 14.6 |
| Penninx et al 2001 (Dutch) | 2847 men and women aged 55-85 mean=70.5(8.7) | 4 | CES-D and DIS | 45 | IHD | Death certificates | Age, sex, education, smoking, alcohol, hypertension, BMI, diabetes, stroke, lung disease, cancer | 7 | 13.6 |
| Cohen et al 2001 (Unite States) | 5564 men and women with hypertension men mean=52.8(9.9) women mean=54(8.8) | 4.9 | Are you now or have you been treated for depression? | 304 | IHD,MI | Medical records and death certificates | Age, race, diabetes, smoking, history of CVD, left ventricular hypertrophy, cholesterol, education, hypertension, SBP, alcohol, BMI, blood sugar, marital status | 7 | 4.6 |
| Wassertheil-Smoller et al 2004 (Unite States) | 93676 women aged 50-79 mean＜65 | 4.1 | CES-D and DIS | 978 | CHD | Self-reports or proxy-reports | Age, race, education, income, BMI, cholesterol, diabetes, smoking, hormone therapy, physical activity, and hypertension | 6 | 28.1 |
| Marzari et al 2005 (Italy) | 2766 men and women aged 65-84 mean≥65 | 4 | GDS | 203 | CHD | Self-reported diagnosis and medical record and official death certificates | Age, marital status, years of schooling ,smoking status , drinking status, depressive symptomatology,  ADL disability level, fibrinogen, platelets  total cholesterol, triglycerides glycaemia, BMI, arrhythmia, hypertension, diabetes, congestive heart failure  claudication, stroke, dementia | 7 | 29.7 |
| Gump et al 2005 (Unite States) | 11216 men aged 35-53 mean＜65 | 18 | CES-D | 1799 | CHD,MI | Death certificates | Age, intervention group, race, education, smoking, blood pressure, alcohol use, cholesterol, and history of cardiac disease | 9 | 19.1 |
| Wulsin et al 2005 (Unite States) | 3634 men and women aged 30-91 mean=52 | 5.9 | CES-D | 83 | CHD | Medical records* | Age, smoking, hypertension, diabetes, BMI, total cholesterol, and alcohol consumption | 8 | 14.1 |
| Kamphuis et al 2006 (Finland, Italy, and the Netherlands) | 799 men aged 70-90 mean≥65 | 7.4 | ZSDS | 93 | CHD | Death certificates | Age, country, education, BMI, smoking, alcohol intake, blood pressure, cholesterol, and physical activity | 8 | 33.4 |
| Ahto et al 2007(Finland) | 660 men and women aged≥64 mean≥65 | 12 | ZSDS | 60 | CHD | Official Death Registry* | Age, occurrence of symptoms of depression, marital status, social status and number of medicines as possible predictors | 8 | 15.2 |
| Whang et al 2009 (Unite States) | 63469 women aged 30-55 mean＜65 | 12 | MHI-5 | 1724 | CHD,MI | Medical records and death certificates or autopsy | Age, beginning year of follow-up, smoking status, BMI ,alcohol intake, menopausal status and postmenopausal hormone use, usual aspirin use ,multivitamin use, vitamin E supplement use, hypercholesterolemia, family history of MI, history of stroke, n-3 fatty acid intake , alpha linoleic acid intake, moderate/vigorous physical activity ,nonfatal CHD during follow-up, hypertension, and diabetes | 7 | 7.9 |
| Davidson et al 2009(Canada) | 1794 men and women aged 18-98 mean=46.3(18.3) | 15514 person-years | CES-D | 152 | CHD | Health register records | Age, gender, and Framingham risk score | 8 | 26.6 |
| Nabi et al 2010(Finland) | 23282 men and women aged 20-54 mean＜65 | 7 | BDI | 203 | CHD | Hospital discharge register or mortality records | Sex, age and education, alcohol consumption, sedentary lifestyle and smoking, obesity, hypertension or diabetes and incident CHD or incident CBVD | 8 | 19.6 |
| Janszky et al 2010(Sweden) | 49321 men aged 18-20 mean＜65 | 37 | Structured clinical interview of ICD-8 | 3189 | CHD, MI | Health register records | Smoking, body length, diabetes, systolic blood pressure, alcohol consumption, physical activity, father’s occupation , family history of coronary heart disease (CHD), and geographic area | 9 | 1.3 |
| Brown et al 2011 (Unite States) | 2728 men and women aged≥60 mean≥65 | 13-16 | CES-D | 727 | CHD, MI | Medical record and National Death Index | Gender, race, diabetes, hypertension, history of smoking, cholesterol, and ideal body weight | 8 | 15.5 |
| Scherrer et al 2011 (Unite States) | 345949 men and women with type 2 diabetes aged 25-80 mean<65 | 7 | ICD-9-CM codes | 11659 | MI | Medical records and register database | Age, sex, race, marital status, insurance type | 7 | 22.4 |
| Majed et al 2012(France) | 9601 men aged 48-64 mean=55 | 10 | CES-D | 647 | CHD | Self-reported diagnosis or confirmed by medical committees | Age; study centers; and socioeconomic factors, including marital status, education level, employment status, physical activity, smoking status, daily alcohol intake, systolic blood pressure, use of anti-hypertensive drugs, BMI, total and high-density lipoprotein cholesterol, treatment for diabetes, and use of antidepressant treatment | 9 | 20.6 |
| Mittag et al 2012(Unite States) | 38947 men and women age≥65 mean≥65 | 2 | Self-report based on three questions: 1) ‘‘In the past year, have you had 2 weeks or more during which you felt sad, blue or depressed; or when you lost interest or pleasure in things that you usually cared about or enjoyed?’’; 2) ‘‘In the past year, have you felt depressed or sad much of the time?’’; 3) ‘‘Have you ever had 2 years or more in your life when you felt depressed or sad most days, even if you felt ok sometimes?’’ | 2424 | IHD | Self-report based on two questions:1)“Has a doctor ever  told you that you had angina pectoris or coronary artery  disease?’’; 2)‘‘Has a doctor ever told you that you had a  Myocardial infarction or heart attack? | Sex and age, hypertension, diabetes, and smoking history | 6 | 50.6 |
| Pequignot et al 2013(France) | 7308 men and women aged 65 mean≥65 | 5.3 | CES-D | 279 | CHD | Self-report and death certificates and autopsy reports | Age, study center, and gender, smoking status, alcohol consumption, high blood pressure, impaired fasting glycaemia or diabetes, hypercholesterolemia, living alone, education level, Mini Mental State Examination (MMSE) score | 9 | 22.7 |
| Sun et al 2013 (Hong Kong of China) | 62839 men and women aged≥65 mean≥65 | 8.4 | GDS | NA | CHD | Register database and international Classification of Disease | Age, education, monthly expenditure, smoking, alcohol drinking, physical activity, BMI, and sex, health status and self-rated health | 8 | 9.6 |
| Gustad et al 2013 (Norway) | 57 953 men and women mean= 47.7(16.3) | 11.4 | (HADS-D) | 2111 | MI | Medical records and National Cause of Death Registry | Calendar age and sex, marital status, education, smoking, physical activity, BMI, total cholesterol, diabetes mellitus, systolic blood pressure | 9 | 3.0 |
| Huang et al 2013 (Taiwan) | 39685 men and women age 20-99 mean<65 | 8.76 | ICD-9-CM clinical diagnosis | 1270 | MI | Medical records | Age, gender, diabetes mellitus, hypertension, hyperlipidemia, alcohol-related illness, obesity, COPD, influenza vaccinations, and cardiology visits | 9 | 20.0 |
| Rahman et al 2013 (Sweden) | 36654 men and women mean=63 | 3.86 | ICD-7,8,9,10-CM clinical diagnosis | 850 | CHD | Medical records and death certificates | Birth year, gender, smoking status, educational level, hypertension, diabetes, alcohol intake and BMI | 8 | 1.6 |
| Brunner et al 2014 (UK) | 10,036 men and women aged 35-55 mean <65 | 24 | GHQ, CES-D | 454 | CHD | Medical records and national mortality register Hospital Episode Statistics* | Age, sex, and ethnicity | 8 | 27.0 |
| Hawkins et al 2014 (United States) | 2537 men and women aged≥ 60 | 15 | CES-D | 678 | MI | Medical records and death certificates | Age, sex, race, diabetes, hypertension, smoking, hyperlipidemia, and excess body weight | 8 | 15.2 |

Age presented the range with Mean(SD); Abbreviations: ADL, Activities of Daily Living; BMI, Body mass index; BDI, Beck Depression Inventory; CES-D, Center for Epidemiologic Studies Depression Scale; CHD, Coronary heart disease; CVD, Cardiovascular disease; CBVD, cerebrovascular disease; DBP, Diastolic blood pressure; DIS, diagnostic interview schedule; GDS, Geriatric Depression Scale; GHQ, General Health Questionnaire; GWB-D, General Well-Being Schedule-Depressed Mood；HADS-D, Hospital Anxiety and Depression Scale; MHI-5, 5-item Mental Health Index; MI, Myocardial infarction; MMPI, Minnesota Multiphasic Personality Inventory; NA, not applicable; OBD, 40 items Obvious Depression Subscale; SBP, Systolic blood pressure; SDS, Zung Self-Rating Depression Scale; SZS , Short Zung Depression Scale

*The outcome for this study is CHD plus MI

**Supplemental Table 2.Subgroup analyses of relative risk of coronary heart disease**

|  | **No of reports** | **Relative risk** | **(95%CI)** | **I^2^** | **P for heterogeneity** |
| --- | --- | --- | --- | --- | --- |
| Controlling smoking in models |  |  |  |  |  |
| Yes | 30 | 1.36 | 1.22 to 1.51 | 61.50% | <0.001 |
| No | 4 | 1.09 | 1.02 to 1.18 | 54.40% | 0.087 |
| Controlling BMI in models |  |  |  |  |  |
| Yes | 22 | 1.41 | 1.23 to 1.62 | 60.10% | <0.001 |
| No | 12 | 1.21 | 1.09 to 1.34 | 77.00% | <0.001 |
| Controlling diabetes in models |  |  |  |  |  |
| Yes | 22 | 1.41 | 1.24 to 1.60 | 59.60% | <0.001 |
| No | 12 | 1.14 | 1.06 to 1.24 | 53.10% | 0.015 |
| Controlling hypertension in models |  |  |  |  |  |
| Yes | 19 | 1.44 | 1.26 to 1.64 | 57.00% | 0.001 |
| No | 15 | 1.15 | 1.06 to 1.25 | 54.00% | 0.007 |
| Controlling physical activity in models |  |  |  |  |  |
| Yes | 10 | 1.24 | 1.06 to 1.45 | 60.60% | 0.007 |
| No | 24 | 1.34 | 1.21 to 1.49 | 75.10% | <0.001 |
| Controlling cholesterol in models |  |  |  |  |  |
| Yes | 13 | 1.30 | 1.11 to 1.52 | 58.30% | 0.004 |
| No | 21 | 1.31 | 1.19 to 1.45 | 76.30% | <0.001 |
| Controlling SES in models |  |  |  |  |  |
| Yes | 20 | 1.20 | 1.10 to 1.31 | 62.80% | <0.001 |
| No | 14 | 1.44 | 1.27 to 1.65 | 46.50% | 0.028 |

Abbreviations: BMI, body mass index; NA, not applicable; SES, socioeconomic status.

**Supplemental Table 3.Subgroup analyses of relative risk of myocardial infarction**

|  | **No of reports** | **Relative risk** | **(95%CI)** | **I^2^** | **P for heterogeneity** |
| --- | --- | --- | --- | --- | --- |
| **Myocardial infarction** | | | | | |
| Primary analysis | 12† | 1.30 | 1.18 to 1.44 | 64.00% | 0.001 |
| **Subgroup analyses for myocardial infarction** | | | | | |
| Sex |  |  |  |  |  |
| Men | 4 | 1.19 | 0.96 to 1.49 | 0.00% | 0.491 |
| Women | 3 | 1.27 | 1.17 to 1.39 | 8.80% | 0.334 |
| Combined | 6 | 1.41 | 1.17 to 1.69 | 79.00% | <0.001 |
| Mean age, y |  |  |  |  |  |
| ≥65 | 3 | 1.39 | 1.03 to 1.86 | 83.70% | 0.002 |
| <65 | 10 | 1.29 | 1.18 to 1.42 | 24.30% | 0.219 |
| Publication year |  |  |  |  |  |
| Before 2005 | 6 | 1.74 | 1.21 to 2.49 | 48.00% | 0.087 |
| 2005-2014 | 7 | 1.26 | 1.15 to 1.38 | 67.20% | 0.006 |
| Duration of follow-up |  |  |  |  |  |
| ≥15 years | 4 | 1.19 | 1.00 to 1.40 | 42.10% | 0.159 |
| <15 years | 9 | 1.36 | 1.23 to 1.50 | 36.40% | 0.127 |
| Study location |  |  |  |  |  |
| Unite States | 9 | 1.28 | 1.13to 1.45 | 67.60% | 0.002 |
| Europe | 3 | 1.35 | 1.06 to 1.71 | 32.00% | 0.23 |
| Asia | 1 | 1.38 | 1.19 to 1.60 | NA | NA |
| Type of depression measurement |  |  |  |  |  |
| Self-reported scales | 9 | 1.30 | 1.13 to 1.49 | 52.10% | 0.033 |
| Clinical diagnosis | 4 | 1.33 | 1.13 to 1.57 | 60.20% | 0.057 |
| Controlling smoking in models |  |  |  |  |  |
| Yes | 9 | 1.23 | 1.10 to 1.39 | 38.10% | 0.114 |
| No | 4 | 1.43 | 1.20 to 1.69 | 64.90% | 0.036 |
| Controlling BMI in models |  |  |  |  |  |
| Yes | 7 | 1.24 | 1.10 to 1.41 | 46.80% | 0.08 |
| No | 6 | 1.38 | 1.17 to 1.64 | 58.60% | 0.034 |
| Controlling diabetes in models |  |  |  |  |  |
| Yes | 8 | 1.28 | 1.12 to 1.45 | 58.90% | 0.018 |
| No | 5 | 1.43 | 1.13 to 1.80 | 55.70% | 0.06 |
| Controlling hypertension in models |  |  |  |  |  |
| Yes | 6 | 1.30 | 1.11 to 1.53 | 68.60% | 0.007 |
| No | 7 | 1.33 | 1.15 to 1.54 | 39.80% | 0.126 |
| Controlling physical activity in models |  |  |  |  |  |
| Yes | 3 | 1.19 | 1.02 to 1.39 | 0.00% | 0.553 |
| No | 10 | 1.35 | 1.20 to 1.52 | 69.00% | 0.001 |
| Controlling cholesterol in models |  |  |  |  |  |
| Yes | 8 | 1.27 | 1.13 to 1.44 | 57.80% | 0.02 |
| No | 5 | 1.47 | 1.10 to 1.98 | 57.10% | 0.054 |
| Controlling SES in models |  |  |  |  |  |
| Yes | 4 | 1.29 | 1.08 to 1.53 | 0.00% | 0.443 |
| No | 9 | 1.31 | 1.17 to 1.46 | 71.20% | 0.001 |

Abbreviations: BMI, body mass index; NA, not applicable; SES, socioeconomic status.

†One article provided RRs for women and men separately, we pooled both risk estimates to obtain one overall estimates for the primary analysis; therefore, there are only 12 reports in the primary analysis.
